# Extra-long treatment of MDR-TB osteomyelitis of humerus due to neurotoxicity from the 2nd-line drugs: a case report

**DOI:** 10.1186/s43162-023-00225-0

**Published:** 2023-06-13

**Authors:** Naser Naser, Habib Abdulla, Husain Kadhem

**Affiliations:** Pulmonary Medicine Unit, Department of Internal Medicine, Salmaniya Medical Complex, Manama, Bahrain

**Keywords:** Pulmonary medicine, Tuberculosis, Osteomyelitis, Radiology

## Abstract

Infection with tuberculosis (TB) still considered a leading infectious cause of death, osteomyelitis TB rare entity, and being extraspinal MDR-TB make it very rare case; most of experience in treating osteomyelitis TB was derived from pulmonary TB experience, and we present a case of humerus MDR-TB that was treated for 5 years, with several interruption due to side effect and other causes.

## Introduction

Global incidence of TB and its mortality worldwide is trending down in the last few years, but still it remains the first leading cause of death among the infectious diseases after COVID-19. India contributes with almost the third of the cases worldwide [1, 2].

Multidrug-resistant tuberculosis (MDR-TB) is a major public problem with estimated global incidence of 3.4% of all new cases of TB not previously treated.

Risk of resistance is increased with HIV patients or who previously treated TB cases reaching up to 18% [3].

Tuberculosis osteomyelitis account around 10% of all TB worldwide, but exact incidence of MDR-TB osteomyelitis is very rare but raising especially in developing countries like India, Russia, and Pakistan and its management facing different challenges in view of using second-line antituberculosis with various side effects [1–3]. MDR-TB in Bahrain is 4%, but our case is first MDR-TB in bone and being extraspinal making it more interesting to share [4].

## Case report

A 28-year-old male patient known to have bronchial asthma and allergic rhinitis presented in February 2014 with complains of fever, productive cough, and shortness of breath for 2 weeks.

He was febrile, and his chest radiograph was showing lower, mid zone consolidation.

He was initially treated as a community-acquired pneumonia with antibiotics. His fever however did not subside, so he had further investigations including sputum culture and acid-fast bacilli smear were negative, and then bronchoscopy with bronchoalveolar lavage was done, turned to be positive acid-fast bacilli smear, and confirm rifampicin resistance, and later culture confirms the MDR-TB as the resistance including rifampicin, isoniazid, ethambutol, and streptomycin.

So, he was started on MDR-TB regimen which include six drugs: amikacin, moxifloxacin, pyrazinamide (PZA), cycloserine (cyclo), linezolid (Lzd), ethionamide (Eto).

However, he was able to continue this regimen for only 3 months, and then he developed severe lower limb sensory motor polyneuropathy. Unfortunately, he took only Eth, cyclo, and para-aminosalycilic acid (PAS) for another 10 months and then ethionamide and cycloserine (due to PAS-induced GI upset) for another 4 months, and after that, the patient chose to stop the treatment after total treatment period of 17 months.

**Fig. 1:**
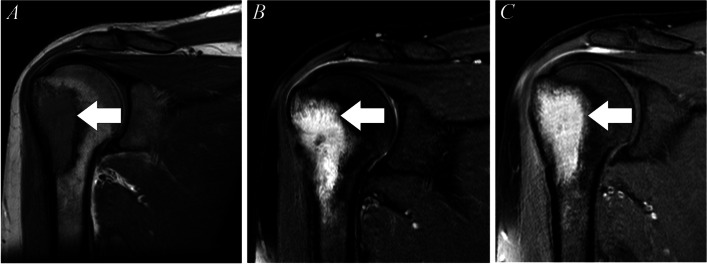
MRI of right shoulder (1) showing ill-defined area of signal abnormality involving the lateral aspect of the greater tuberosity (arrow). It demonstrates a low signal intensity on T1 (**A**) and a bright signal on the T2 fat-saturated images (**B**). On the post-contrast image (**C**), the majority of the intramedullary component demonstrates thick peripheral rim enhancement and areas of solid enhancement, indicating osteomyelitis

Two months later, he started to complain of right shoulder pain, tenderness with right shoulder pain, and with limitation of movement for 4 months. MRI shoulder showed abscess-like lesion in the proximal humerus (Fig. 1). At this stage, he denied any respiratory symptoms, while his chest X-ray showed resolution of right lower lobe consolidation.

He got admitted for incision and drainage. The bone was found to express frank pus which was sent for TB-PCR which came also positive from the pus.

**Fig. 2:**
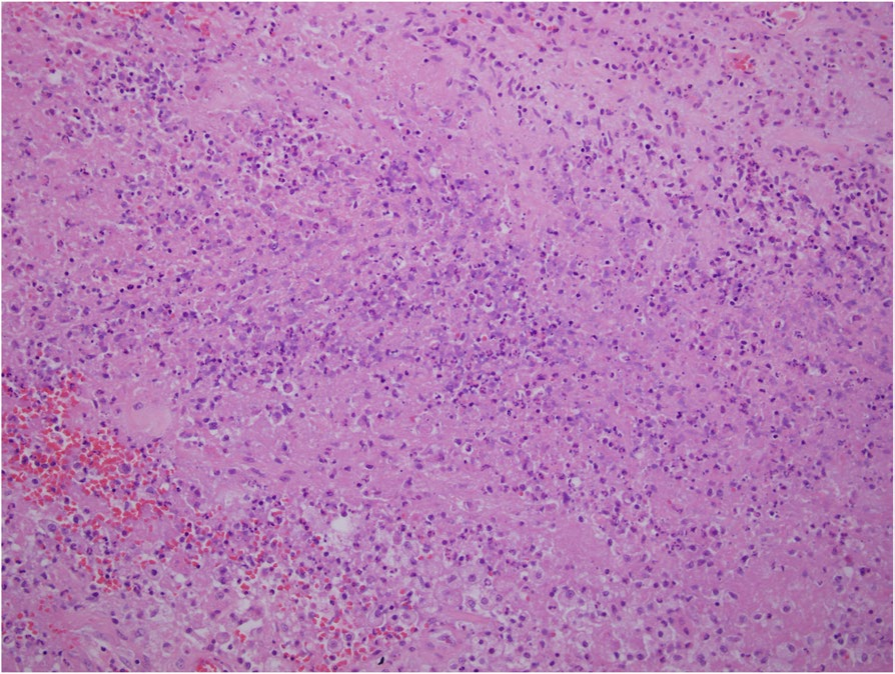
The section shows granulomatous inflammation with foci of necrosis in the center

Biopsy specimen of the bone revealed necrotizing granulomatous inflammation and abscess formation (Fig. 2). After a discussion between the Infectious & respiratory teams which decided to resume second line anti TB and due to the high possibility being even of XDR-TB especially that quinolone sensitivity was not available.

Moreover, a decision was made to treat the patient with the initiation phase of treatment was given for 4 months (6 drugs: Mfx, Lzd, capreomycin, cyclo, PZA, Clr); he was instructed for good compliance.

He was able to be on this regimen only for 4 months as he developed again severe peripheral neuropathy in his lower limbs and hands despite using high dose of vitamin B6, i.e., pyridoxine (200 mg daily) plus symptomatic treatment with gabapentin and carbamazepine. So Capreomycin, Moxifloxacin & Linezolid were suspended then he was continued on {PZA, PAS,, Cyclo,Co-amoxiclav (Clav) & Bedaquiline (Bdq)} was started then continued for 1 year only because of shortage in cycloserine supply it was discontinued so he remain on {PZA, PAS, Bdq, Clav & Clarithromycin (Clr)} which is considered relatively weak regimen although the patient was advised to resume either linezolid or fluoroquinolone but he was reluctant because of neurotoxicity as well as no other options of second line anti TB regimen was available.

**Fig. 3:**
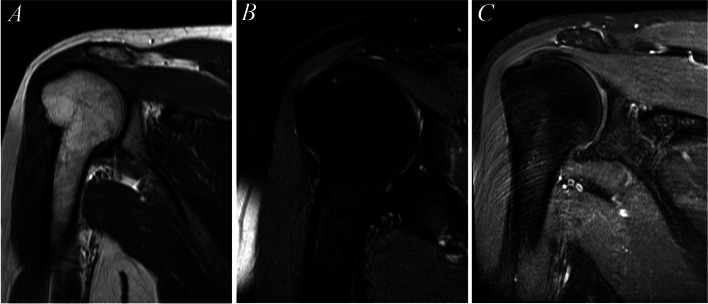
Follow-up MRI (**A**) resolving of proximal humeral intramedullary signal abnormality and appears organized in appearance with no abnormal high signal intensity in T2 (**B**) or abnormal enhancement (**C**). The picture is in keeping with resolving of the osteomyelitis with granulation tissue. No new changes or areas worrisome for infection

The last second-line regimen mainly depends on three drugs including PZA, PAS, and Bdq according to the recent WHO guidelines 2020 in addition to old two drugs which is Clr and Clav which is considered no longer recommended in prolong MDR-TB treatment [4], so he was continued on this regimen, but for longer duration guided by radiological imaging, he completed another 4 years with serial follow up of MRI humorous which showed dramatic response of the size of the osteomyelitis infection (Fig. 3).

In view of suboptimum continuation phase, so total treatment was extended to almost 5 years, and it was discontinued with follow-up MRI showing dramatic improvement after 14 months from stopping the treatment without any signs of recurrence.

## Discussion

Rifampicin-resistant TB is caused by *Mycobacterium* *tuberculosis* strains resistant to rifampicin. These strains may be susceptible or resistant to isoniazid (i.e., MDR-TB) or resistant to other first-line or second-line TB medicines, while XDR-TB is defined as MDR-TB which is resistant extended to one of the injectable second-line drugs and fluoroquinolone.

Its prevalence varies from one region to another, most cases reported in India, Russia, and Pakistan [1]. However, in one study in India, incidence of TB in bone with majority of the cases tuberculous spondylitis is 54%, while the rest were extraspinal, among all extraspinal MDR-TB was documented in 3.7%, and XDR-TB was documented in 0.4% of extraspinal tuberculosis [5, 6].

Diagnosing of MDR-TB osteomyelitis frequently delayed as it needs careful interpretation of radiological imaging, and most of the cases need surgical intervention to reach the diagnosis like our case; hence, the use of Xpert MTB/RIF assay in tuberculous bone also increases the sensitivity of earlier diagnosis of MDR-TB in bone to 82% [7].

Although our case was showing typical necrotizing granulomatous inflammation (Fig. 2), some of the TB osteomyelitis bone biopsy may show only chronic nonspecific osteomyelitis [2].

The medical treatment of MDR-TB in bone has no international guidelines for it in terms of the drug of choice, the optimum duration but most of the cases treated as pulmonary MDR-TB with duration of 18–24 months or longer depends on the clinical response, and the tolerance of the second-line anti-TB [7].

Multidisciplinary team approach recommended as more than 60% of the cases require surgical intervention to help in diagnosis, treatment, and follow-up especially to deal with various side effects of medication.

Our patient underwent debridement of his proximal humerus abscess, which has significantly reduced the burden of the infection.

During medical treatment, our patient had a deleterious neurotoxicity and lack availability of other options of treatment which is less neurotoxic like clofazimine or delamanid making it more difficult.

Another major obstacle that the sensitivity of the second line anti-TB drugs was not available, especially that our case is resistant to one of the injectable (Strptomycin) making possibility of being XDR-TB is higher

Introducing bedaquiline [8] throughout the treatment period makes us able to cure the patient, although the duration of the therapy was extended to 5 years.

The latest WHO announcement regarding the MDR-TB treatment emphasizes on the role of bedaquiline containing regimen, with the aim of shorter and all oral treatment course [9, 10].

Although this announcement came after we finish our patient treatment, the optimum duration was guided by clinical condition, periodic MRI follow-up to ensure no more residual disease in the bone, and last, MRI was done after 1 year from stopping medication.

In MDR-TB osteomyelitis, physician should be trained well to deal with multiple side effects to balance between tolerance of the side effects and the strength of the second-line regimen.

In conclusion, data on the treatment of MDR/RR TB osteomyelitis is lacking; however, most observational studies and case reports point toward following the same therapeutic guidelines for the treatment of MDR pulmonary TB, and new data is coming offering the all-oral regimen.

## Abbreviations

TB: Tuberculosis
MDR: Multidrug resistant
XDR: Extremely drug resistant
Bdq: Bedaquiline
PZA: Pyrazinamide
Eth: Ethionamide
PAS: Para-aminosalycilic acid
Clav: Co-amoxiclav
Clr: Clarithromycin
Cycl: Cycloserine
